# Field demonstration analyzing the implementation of individual animal electronic identification and genetic testing in western range sheep flocks

**DOI:** 10.1371/journal.pone.0290281

**Published:** 2023-08-23

**Authors:** Julie A. Finzel, Austin R. Brown, Roselle C. Busch, Morgan P. Doran, John M. Harper, Daniel K. Macon, Rebecca K. Ozeran, Morgan R. Stegemiller, Karissa Isaacs, Alison Van Eenennaam

**Affiliations:** 1 University of California Cooperative Extension, Kern County, University of California Agriculture and Natural Resources, Bakersfield, California, United States of America; 2 Department of Animal Sciences, University of California, Davis, California, United States of America; 3 University of California Cooperative Extension, Solano County, University of California Agriculture and Natural Resources, Woodland, California, United States of America; 4 University of California Cooperative Extension, Mendocino County, University of California Agriculture and Natural Resources, Ukiah, California, United States of America; 5 University of California Cooperative Extension, Placer County, University of California Agriculture and Natural Resources, Auburn, California, United States of America; 6 University of California Cooperative Extension, Fresno County, University of California Agriculture and Natural Resources, Fresno, California, United States of America; 7 Department of Animal, Veterinary, and Food Sciences, University of Idaho, Moscow, Idaho, United States of America; 8 Superior Farms, Denver, Colorado, United States of America; Ain Shams University Faculty of Agriculture, EGYPT

## Abstract

Adoption of electronic identification ear tags (EID) and DNA testing by commercial range sheep producers in the Western United States has been low, despite the availability of these technologies for over a decade. Jointly, these technologies offer an approach to provide individual animal performance data to improve flock health, genetic and reproductive management. This project involved a collaboration with five California sheep producers representing a broad geographic range, varying levels of pre-project EID adoption, and diverse operational practices. Tissue samples were collected from, and ear EIDs were placed in, a total of 2,936 rams and their potential lambs. We partnered with a commercial packing company, Superior Farms, to genotype the animals. Superior Farms used a targeted genotyping panel to assign parentage, and link individual animal identification (ID) to camera-graded carcass measurements. This enabled the collection of individual progeny carcass data and provided insight into sire performance, providing for the within-flock identification of prolific sires that were producing lambs with significantly more saleable meat as compared to their flock mates. Overall, almost 91% of lambs were successfully matched to their sire, and prolificacy ranging from 0–135 lambs per ram. There was as much as an $80 difference in the average edible product from camera-graded carcasses derived from lamb groups sired by different rams. A partial budget analysis modeling investment in an EID system coupled with an autodrafter and scale to collect individual weights and improve labor efficiency during processing, and a sheep flip chute to improve worker safety during foot trimmings, yielded a greater than 7:1 return on investment over a five-year time frame. Ideally, the data collection enabled by EIDs and DNA testing would feed into data-driven genetic evaluation programs to enable selection for more productive and profitable animals, and allow the US sheep industry to accelerate the rate of genetic improvement.

## Introduction

Rates of genetic improvement and potential production efficiencies in the Western range sheep population of the United States are substantially lower than what is theoretically possible and considerably slower than what has been achieved by other livestock species. Genetic improvement requires data recording on individual animals, such as weighing lambs at weaning and recording metrics of ewe performance. Producers in extensive production systems own an outsized proportion of the national sheep inventory, as many large commercial sheep operations average well over 1,000 animals [1], which makes it difficult to track the performance of individual sheep. As a result, the main husbandry practices in many large Western range sheep operations are group-oriented and the opportunity for individual animal recording is limited by the scarcity and cost of labor [2].

In large flocks, management is geared towards the average performing individuals, with occasional focus on low-performing individuals, e.g. extended breeding seasons for ewes that didn’t breed in the first 3 cycles, or supplemental feeding for animals with a low body condition score. Generally, producers only obtain data on their flock in aggregate. Using weaning weights as an example, a producer knows the weight of each load of lambs shipped, but not the weight of each lamb in the load.

Electronic identification (EID) ear tags facilitate rapid and accurate data collection that can be used for informed decision making on several aspects of flock management, such as breeding (i.e. selecting individuals with desirable genetic traits) and health (i.e. lameness, particularly with respect to culling repeatedly lame sheep) [3]. These tags can be read with the use of scanners and then data can be uploaded into a producer`s database directly in the field. This reduces potential errors while increasing time management efficiency by reducing the need for redundant data entry. When individual animal identification is combined with records on economically important traits, poorly performing animals can be removed from the flock to increase overall flock performance. The introduction of EIDs has the potential to decrease the time and labor needed to track performance traits of individual animals within a flock. The use of EID has proven to be cost-effective in sheep production systems in other countries [4, 5]. However, despite the known benefits, Western sheep producer adoption of this technology remains low.

Genomic selection has been widely adopted in dairy cattle breeding programs, yet only a few countries have implemented genomic selection for sheep [6]. This is because the accuracy of sheep sire genetic evaluations tend to be low due to the limited use of artificial insemination making it difficult to compile reference populations, and because the cost of genotyping is high relative to the value of the animal [7]. As a result, genomic selection is not always cost-effective in sheep [5]. In major sheep-producing countries including Ireland, New Zealand, and Australia multi-tiered breeding structures and selection based on estimated breeding values (EBV) are common [8]. In these countries, several studies have modeled the cost-benefit of genotyping and phenotyping in the commercial sheep sector, where the data are used to improve the accuracy of genomic-enhanced EBV (GEBV) [9, 10]. Few papers look at the value of targeted genotyping panels to identify parentage and single nucleotide polymorphisms (SNP) markers associated with commercially important traits in the absence of an overarching comprehensive genetic evaluation program. Identifying parentage allows producers to determine which rams are the most active breeders in their flock. Genetic testing combined with individual animal performance records could also be used to create within-flock genetic merit estimates to identify the best performing rams.

In this demonstration project, we collaborated with five producers throughout California to assess how EIDs and genetic testing could inform ram selection and provide value to producers. Our objective was to work with these producers to determine how the data collected from these technologies could provide value to commercial ranches. Familiarity and use of EID tags on ranches at the start of the research varied, allowing for a qualitative reflection on the participant`s perceptions of the usefulness of this technology. Moreover, we partnered with Superior Farms (Dixon, CA) to acquire much of the data analyzed in this project. Superior Farms is an employee-owned company and the largest lamb processor in the United States. They are headquartered in Sacramento, CA with production facilities in Dixon, CA and Denver, CO. Collaborators marketed their lambs to Superior Farms, and they were able to aid in the following aspects of this project: 1) genotype the animals using a targeted genotyping panel, 2) assign parentage, and 3) link individual animal ID to camera-graded carcass measurements. This enabled the collection of individual progeny carcass data and provided insight into sire performance, providing for the within-flock identification of prolific sires that were producing lambs with significantly more saleable meat as compared to their flock mates.

Thanks to our collaborators, we were able to incorporate the following four questions into a calculated consideration as we sought to outline a plan for in-field implementation of EID tags. 1) What new or additional expenses will be incurred? 2) What current costs will be reduced or eliminated? 3) What new or additional income will be received? And 4) What current income will be lost or reduced?

## Materials and methods

The five producers enrolled in this project represented a broad geographic range from Northern California to the southern San Joaquin Valley, varying levels of pre-project EID adoption, and diverse operational practices including targeted grazing. All producers maintained wool breed ewe flocks with one small flock (<100 breeding ewes) and four large flocks (>2000 breeding ewes). Cooperating producers were interviewed at the beginning of the project to obtain data on their operation and estimates of typical production parameters and costs. Ranches were randomly assigned with a letter (A-E) for means of anonymously reporting the flocks’ data. Tissue samples were collected from rams and lambs for DNA testing and each animal sampled received an EID ear tag that was scanned using an electronic reader. The EID tag uniquely identifies an animal and all of its pertinent management information can then be associated with that unique ID. The timestamp from the reader after scanning provided a time estimate for the added steps to collect genetic samples and apply ear tags during processing. This was important when evaluating potential time savings for producers.

Rams were sampled in the spring of 2019 using tissue sampling units from AllFlex (Kenilworth, NJ, USA). Lambs from the four large flocks (2000+ breeding ewes) were sampled between November of 2019 and February 2020 using a combination of docked tails and ear notches. For the small flock, the producer collected their own samples using tissue sampling units in the spring and summer of 2020. All fresh tissue samples were stored in a freezer prior to submission; tissue sampling units are shelf stable at room temperature. The study protocol was approved by the UC Merced Institutional Animal Care and Use Committee (protocol number: AUP19-0005).

Tissue samples were submitted to Superior Farms`(Dixon, CA, USA) Flock54_SM_ genetic testing program which they offer commercially to the sheep industry [11]. Flock54_SM_ is a SNP panel containing 956 markers including over 100 markers for parentage analysis [12] and around 60 markers associated with disease and/or production traits, and approximately 800 markers common to the Ovine 50K chip and evenly spaced throughout the genome to facilitate imputation to higher density SNP chips. Flock54_SM_ generates reports of genotypes and parentage assignments for easy interpretation of the data for producers. It also includes selection points for tracing susceptibility to diseases, including scrapie and ovine progressive pneumonia (OPP).

Tissue samples were submitted in two groups. The first group was submitted in May of 2020 and the second group was submitted in October of 2020. In this study, the dam’s genotype was not collected so population genotype frequencies were estimated from the genotypes of all rams and lambs collected. Paternity based on SNPs was determined by comparing the genotypes of all potential sires against each lamb`s genotype. An exclusion was recorded when a ram and a lamb had no allele(s) in common at an identified marker. Genetic data from offspring had poor rates of paternity matching, and in those instances, we utilized a software package called SireMatch to obtain paternity results based on the raw SNP data (E. J. Pollak, personal comm.). SireMatch uses a likelihood-based method to compute a probability that a putative sire is the correct sire given genotypes of the lamb and all possible sires [13]. All parentage analyses included over 100 parentage SNPs. No more than three exclusions between the SNP genotype of a potential sire and offspring were allowed and paternity was listed as unknown if no ram met the criteria of 2 or less exclusions. There were no cases where 2 or more rams were identified as a potential sire for a given lamb.

The SNP data was analyzed with the SNP & Variation SuiteTM version 8.7.2 (Golden Helix, Inc., www.goldenhelix.com). Markers with an identification rate <0.9, minor allele frequency <0.01 and any markers on the X chromosome were removed. A total of 793 markers were utilized to calculate eigenvalues. A principal component analysis (PCA) plot was created by plotting the largest two eigenvalues and each sample was color coded by ranch.

Study ram lambs were processed at Superior Farms`slaughterhouse facility. Carcass data was collected on these lambs using a VSS2000 camera grading system (e+v, Oranienburg, Germany). Each lamb`s carcass metrics were linked to its correlating EID by scanning the tag provided at the beginning of the project. Superior Farms measured hot carcass weight, and the camera grading system was used to predict yield grade, quality grade, common cuts, Ovine Cutability Calculation (OCC) and OCC yield. OCC predicts the weight of primal cuts less trim of fat and bone. OCC is estimated with an algorithm that uses digital photographs captured at rail speed. OCC yield is the ratio of that weight over the carcass weight, expressed as a percentage. A higher OCC yield indicates that an animal yielded more raw muscle product per pound of carcass after being trimmed. We also calculated a dollar difference in edible product using the USDA Ag Marketing Service Report price to find a net carcass cutout value ($/kg * average flock sire group deviations for OCC). Each ram’s offspring were evaluated as a "progeny group" and the deviation of the progeny group mean from the overall mean was calculated. The average number of lambs per sire born and the sire group mean for OCC at each ranch, and the mean for carcass attributes for each ranch were compared using a one-way analysis of variance.

A 4-year partial budget simulation was developed to demonstrate the potential benefits and costs of investing in an EID tag system. Cost and returns estimates were based on costs shared by one of the participating producers based on their experience using EIDs. We modeled a scenario in which a typical western range sheep operation invested in multiple pieces of equipment to achieve the goals of: 1) better informed genetic selection of replacement breeding females; and 2) improved labor efficiency and safety. Specific objectives were to improve overall flock foot health and increase kilograms of lamb weaned per ewe exposed.

For this analysis we assumed a base flock of 3,000 breeding ewes with a beginning lambing rate of 90%. Ewes remain in the flock for an average of five years indicating a 20% annual replacement rate. We calculated costs and returns over a four-year period beginning at day zero. Due to variable tax and shipping rates, we did not include tax or shipping on any purchased items. Given the instability in the inflation rate, we opted to forego any calculation of net present value. The partial budget analysis was conducted in Excel.

## Results

Table 1 provides a detailed profile of the five cooperating producers enrolled in the project. Ranches A, B, and E ran traditional white face maternal range ewes; typically crossed with Suffolk sires to generate terminal lambs for market, although for Ranch B in this trial we only sampled lambs from the maternal band. Extensive Western grazing systems have traditionally utilized Rambouillet, or its derivative breeds, to produce a white face ewe which produces a fine-diameter wool and denser fleeces that capture higher sale prices than wool traditionally produced in mixed crop–livestock systems where the focus is more on increased lamb production per ewe [2]. Ranch D ran US Meat Animal Research Center crossbred white face range ewes [14] crossed with terminal Texel x Suffolk rams, and marketed their lambs early to a non-traditional market. Ranch C was a smaller producer who used Blueface Leicester-cross (Mules) and Shropshire sheep to produce lighter lambs for sale into a non-traditional market.

**Table 1:** Summary of sheep producers participating in the study and description of each operation.

	Ranch A	Ranch B	Ranch C	Ranch D	Ranch E
**Flock size**	Large	Large	Small	Large	Large
**Breeds of Rams**	Black and White-face	Black and White-face	Black-face	Texel x Suffolk	Black and White-face
**Average Lamb Crop**	145%	130%	140–150%	145–150%	115%
**Length of Breeding Season**	75 days	75 days	62 days	120 days	185 days
**Avg. Weaning Weights (Kg)**	39–50	32–43	27 (~3 mos.)	48–50 (4–7 mos.)	50–52
**Currently uses EID’s**	Yes	Yes–on maternal flock	Yes–on all animals	No	No
**Traits tracked with EID’s**	None*	Health, pregnant vs. open, twins, wool microns	Disease, BCS, vaccines, wormers, dam of lambs	None*	None*
**Methods to Measure Maternal Traits**	Informal observation	Tag problem ewes	Yes—EZ Care System	Mark poor mothers	No
**Pregnancy Check**	No	Yes–separate singles and doubles	No	Yes	No
**Track Twins**	Yes	Yes	Yes–EID’s	No	Yes–paint brand
**Yearly maintenance cost of a ewe**	$113	$120	$99 (no labor costs)	$210	$150
**Producer Comments**	Major focus on twinning	Goal of breeding program is to eliminate the bottom (10–15%) of the flock	EID data are more actionable	Focus on culling bottom 1/3 of flock	N/A^t^

* None indicates no traits tracked with EIDs

^t^N/A No response

**Fig 1:**
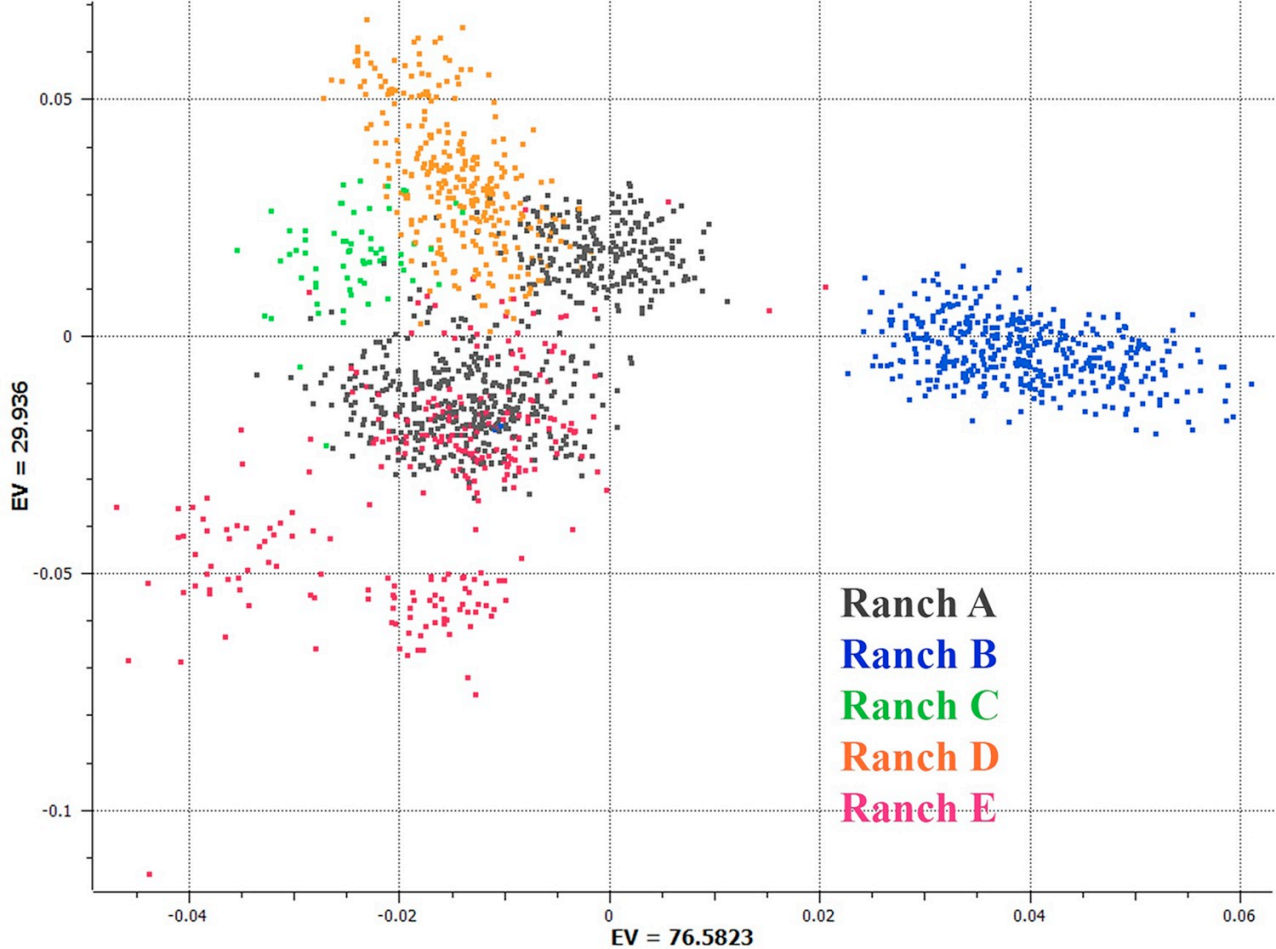
PCA plot. Principal component analysis of the single nucleotide panels collected from the five ranches. Ranch A = Black, Ranch B = Blue, Ranch C = Green, Ranch D = Orange, Ranch E = Magenta.

A total of 2,936 tissue samples from rams and lambs were collected and submitted to Superior Farms (Dixon, CA, USA) for genetic testing. A total of 632, 422, 64, 302 and 273 animals from Ranches A-E, respectively, were included in the Principal Component Ananlysis (PCA) plot (Fig 1). Each dot represents an individual and the distance between the dots reflects the genetic relatedness between them. Individuals with similar genetic backgrounds are closer together on the plot, while those with more dissimilar backgrounds are further part. Animals within a flock can be seen to cluster together, and the genetic variation between the different flocks can be visualized. Ranch B can be seen to cluster distinctly from the other flocks as only samples from their maternal band of purebred Rambouillet sheep were submitted, whereas other ranches submitted samples from their terminal band which typically featured black-faced Suffolk rams.

**Fig 2:**
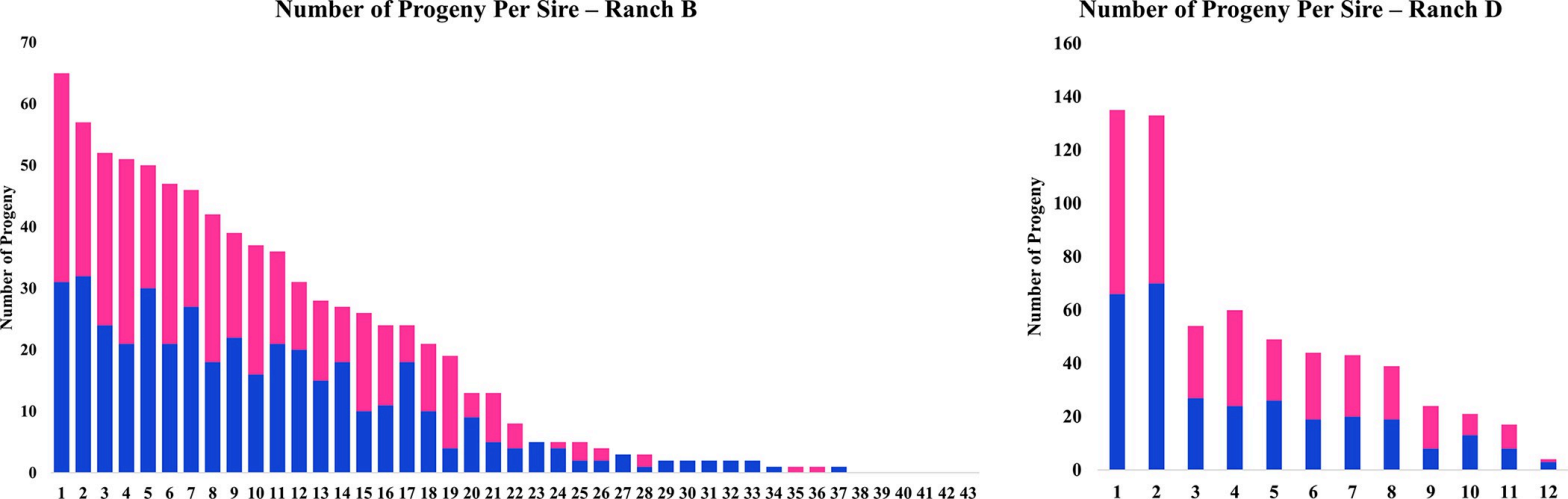
Number of progeny per sire. Progeny born per sire at 2a) Ranch B and 2b) Ranch D. Each number on the x-axis represents a ram while the y-axis show the total number of progeny a ram sired. Blue indicates male lambs & pink indicates female lambs.

Paternity assignments were determined by excluding rams that could not be a lamb’s sire based on their lack of shared genetic markers. Assignment results varied from ranch to ranch, but, overall, 90.8% of lambs were successfully matched to their sire (Table 2). Due to funding limitations, only male lamb samples from Ranch A, and only female lamb samples from Ranch E were submitted for genotyping. Ranch C and Ranch D had the highest match rates, with 100% of lambs matched with their sire. Ranch A had 91% of lambs matched to sires, and Ranch B had 96% of lambs matched to sires. Ranch E had the lowest match rate at 67% of lambs matched to sires. In discussion with Ranch E, it was learned that clean-up rams that were turned out with the flock had not been DNA sampled for genetic analysis. There was a significant ranch effect on the average progeny number per ram (p-value < 0.05) with the ranch having the lowest ram to ewe ratio (1:10) having the lowest average progeny number per ram, and the ranch with the greatest (1:50) having the highest. There was also a large range in the number of lambs per sire (0–135) within each ranch (Table 2). Of note, there was large variation in the ram to ewe ratio between ranches. The ram to ewe ratio was loosely associated with the number of progeny per sire, averaging 23 lambs across all ranches, however, there were some highly prolific rams on ranches with a low ram to ewe ratio. For example, on Ranch A with a ram to ewe ratio of 1:10, one ram sired 66 male progeny. Assuming this represented half of his progeny as female lambs were not genotyped, this suggested he likely sired ~ 122 lambs. Ranch D with a ram to ewe ratio of 1:50, had two rams that sired over 100 lambs with the top producing ram siring 135 lambs (Fig 2).

**Table 2:** Summary of paternity assignment analysis.

	Ranch A	Ranch B	Ranch C	Ranch D	Ranch E	Summary of all Ranches
**Ram to Ewe Ratio**	1:10	1:35	1:13	1:50	1:40	1:33 (Average)
**Total Lambs**	669 (Males only)	829	80	622	222 (Females only)	2422
**Total lambs w/an identified sire**	606	796	80	622	149	2253
**Sires with lambs /Total sires sampled**	62**/**82	37**/**43	6**/**7	12**/**12	11**/**25	128
**Parentage ID rate, %**	91	96	100	100	67	90.80 (Average)
**Standard Deviation**	12.5	19.8	12.6	41.7	25.0	22.32 (Average)
**Average progeny per sire**	9.9 (Males only)	21.5	13.3	51.9	20.1 (Females only)	23.34 (Average)
**Max Progeny from one sire**	61 males	65	33	135	59 females	70.69 (Average)
**Minimum progeny from sires with offspring**	1 male	1	1	4	2 females	1.80 (Average)
**Median progeny amongst all rams with offspring**	4 males	19	11.5	43.5	4 females	16.40 (Average)

Flock54_SM_ generates reports of susceptibility to diseases, including scrapie and OPP. Flock54_SM_ reports OPP susceptibility on a scale from one to ten. A higher score indicates a lower susceptibility to the OPP virus. The average score in all flocks was found to be 5.88, with a range of 1–8 in individual animals. A genotype for Scrapie, a prion-derived wasting disease, was also reported with RR being very resistant, QQ being most susceptible, and the heterozygous QR variant being much less susceptible. The frequency of the two alleles in this study was approximately equal, and a typical Hardy-Weinberg equilibrium ~25% RR, ~50% QR, and ~25% QQ was observed in this study across all ranches. Additionally, a measure of fecundity was evaluated at the growth differentiation factor 9 (*GDF9*) gene. In this study thirty-five lambs came back with a positive result. Six of these lambs came from Ranch A and twenty-eight came from ranch D and were heterozygous carriers (+/-). One lamb came back with a Positive2 result, meaning it was homozygous (+/+) for the *GDF9* allele, on ranch E. Three lambs from Ranch D also came back with a genotype result associated with muscle hypertrophy. Ranch D showed some prevalence of Myostatin in the lambs tested because five of the sires in their stud battery were carriers for the Myostatin mutation which is found naturally in the Texel sheep breed. The G-to-A transition in the 3′UTR of *Gdf8* in Texel sheep results in microRNA-mediated silencing of *Gdf8* translation which results in muscle hypertrophy in Texel sheep. Texel sired progeny show intermittent heavily muscled phenotypes associated with muscular hypertrophy.

Table 3 further highlights some of the unique single gene genotypes present in the progeny tested across all five ranches.

**Table 3:** Summary of progeny genotype results.

	Ranch A	Ranch B	Ranch C	Ranch D	Ranch E
**Average OPP Resistance Score**	6.44	6.18	4.84	5.42	6.51
**Myostatin Carrier**	0	0	3	0	0
**GDF9 Fecundity +/+**	0	0	0	0	1
**GDF9 Fecundity +/-**	6	0	28	0	0

Unfortunately, supply chain disruptions due to the Covid-19 pandemic resulted in the ram lambs from only two ranches being processed at Superior Farms. In total, only 524 male lambs were processed and graded at Superior Farms. Table 4 shows a summary of average carcass traits by ranch, including p-values from one-way analysis of variances for each trait. Results indicate a significant difference in eight of the eleven traits.

**Table 4:** Summary of average carcass traits from lambs processed from Ranch A and Ranch B.

Ranch A	HCW (kg)	Yield Grade	Breast (kg)	Rack (kg)	Shoulder (kg)	Legs (kg)	Loins (kg)	Neck (kg)	Trotters (kg)	OCC (kg)	OCC Yield (%)
Average	33.323	2.700	3.968	3.656	8.184	10.748	3.752	0.753	1.475	21.946	66.478
n	209	205	205	205	205	205	205	205	205	205	205
Std Dev	4.62	0.50	0.98	0.76	1.50	2.05	0.65	0.15	0.23	4.23	9.38
**Ranch B**											
Average	34.508	2.700	4.294	3.954	8.605	11.147	3.793	0.821	1.533	22.988	67.341
n	315	315	315	315	315	315	315	315	315	315	315
Std Dev	5.28	0.50	0.90	0.63	1.15	1.63	0.38	0.13	0.12	3.37	1.14
**ANOVA**	**<0.01**	0.407	**<0.001**	**<0.001**	**<0.001**	**<0.05**	0.361	**<0.001**	**<0.001**	**<0.01**	0.106

**Fig 3:**
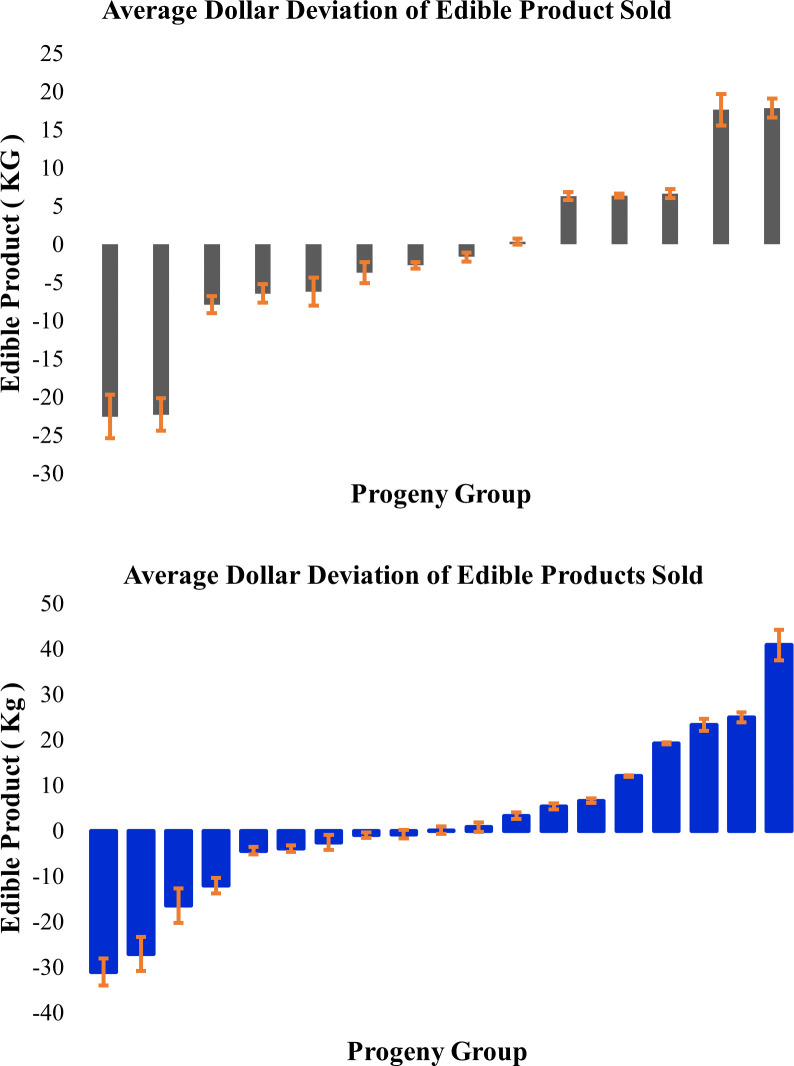
Dollar difference in edible product. 3a) Ranch A, and 3b) Ranch B sire family deviation in edible product for rams; each bar represents a ram with at least 5 progeny.

The carcass data obtained from the sires`progeny from Ranch A and B was used to calculate average sire family deviations for the rams (Table 5). The dollar difference in edible product used a value of OCC *13.39 $/kg. The graphs in Fig 3 illustrate the varying saleable product that resulted from lambs produced by different sires at Ranch A (Fig 3A) and B (Fig 3B). There was a significant sire effect (p-value < 0.05) on average OCC within each ranch, meaning some sires were producing offspring with significantly more saleable meat.

**Table 5:** Average sire family deviations for the rams with at least five progeny from Ranches A and B.

Sire ID	Hot WT. CG Diff. (KG)	Hot Weight STDEV.	Yield Grade CG Diff.	Yield Grade STDEV.	OCC CG Diff. (KG)	OCC STDEV.	OCC Yield CG Diff.	OCC Yield STDEV.	$ of Edible Product	$ of Edible Product STDEV.
**Ranch A**										
***A***	-1.63	1.06	-0.36	0.27	-0.91	0.61	0.71	0.29	-12.15	8.17
***B***	-0.87	0.72	-2.24	1.11	-0.54	0.45	0.35	0.13	-7.29	6
***C***	1.6	0.35	0.2	0.01	0.93	0.19	-0.28	0.14	12.39	2.55
***D***	1.7	0.39	0.26	0.01	0.98	0.21	-0.28	0.14	13.06	2.83
***E***	0.61	0.05	0.36	0.05	0.05	0.15	-0.99	0.4	0.67	2.06
***F***	4.41	1.39	0.5	0.1	2.59	0.81	-0.75	0.31	34.67	10.79
***G***	-0.64	0.49	-0.16	0.14	-0.24	0.25	0.48	0.15	-3.22	3.31
***H***	-4.6	1.78	-0.33	0.19	-3.31	1.26	-0.84	0.3	-44.33	16.82
***I***	-0.99	0.58	-0.33	0.19	-0.94	0.47	0.43	0.12	-12.63	6.25
***J***	-2	0.87	-0.44	0.21	-1.16	0.51	0.65	0.19	-15.55	6.85
***K***	1.16	0.12	-0.08	0.09	0.94	0.14	0.67	0.18	12.57	1.86
***L***	4.29	1.02	0.54	0.09	2.62	0.62	-0.51	0.16	35.1	8.36
***M***	-5.22	1.66	-0.3	0.15	-3.27	1.03	0.13	0.02	-43.78	13.84
***N***	-0.47	0.34	0.06	0.05	-0.4	0.24	-0.16	0.06	-5.4	3.2
***Ranch B***										
***A***	2.67	0.87	0	0	1.74	0.26	-0.1	0.04	23.32	14.12
***B***	-1.46	0.64	0.02	0.01	-1.23	0.8	-0.3	0.11	-16.46	0.86
***C***	4.71	1.54	0.58	0.2	3.06	0.71	-0.03	0.01	40.93	19.43
***D***	-0.55	0.29	-0.01	0	-0.19	0.39	0.54	0.17	-2.55	3.63
***E***	2.67	0.73	0.14	0.04	1.87	0.26	0.24	0.08	25.02	12.35
***F***	-2.93	0.99	-0.16	0.05	-2.02	0.92	-0.22	0.07	-27.02	3.92
***G***	1.38	0.31	0	0	0.9	0.04	-0.06	0.02	12.08	7.87
***H***	-0.16	0.14	0.04	0.01	0.06	0.27	0.54	0.15	0.85	4.13
***I***	0.55	0.05	-0.09	0.02	0.4	0.17	0.14	0.04	5.4	5.19
***J***	-3.46	0.99	-0.11	0.03	-2.32	0.87	-0.1	0.03	-31.03	4.39
***K***	-1.51	0.48	-0.12	0.03	-0.9	0.5	0.36	0.09	-12.02	0.52
***L***	0.56	0.05	0.01	0	0.25	0.21	-0.35	0.09	3.34	4.48
***M***	0.88	0.13	0	0	0.49	0.14	-0.15	0.04	6.62	5.33
***N***	-0.32	0.17	-0.01	0	-0.05	0.28	0.42	0.11	-0.73	3.32
***O***	0.11	0.06	-0.05	0.01	0.01	0.25	-0.21	0.05	0.12	3.43
***P***	-0.33	0.15	-0.01	0	-0.33	0.29	-0.36	0.08	-4.37	2.06
***Q***	-0.68	0.21	-0.17	0.03	-0.29	0.27	0.49	0.1	-3.89	2.03
***R***	0.1	0.05	0.06	0.01	-0.07	0.22	-0.41	0.08	-0.91	2.62
***S***	2.24	0.36	0.18	0.03	1.44	0.08	-0.13	0.03	19.25	6.4

The partial budget is detailed in Table 6. We estimated the cost of two EID tag readers at $2,500 each and EID ear tags for the entire base flock at $1 per tag and four tag applicators, $30 each. We modeled purchasing tags for all retained replacement females, 600 head per year for four years. We modeled an autodrafter with a scale, estimated cost $13,800, to improve labor efficiency during processing and improve feedback on animal size and performance. To further enhance labor efficiency and improve worker safety during foot trimmings a sheep flip chute was included for $3,000. The management software used to analyze the individual animal performance records was estimated at $0.75 per head per year. We valued management time to utilize the software and make management decisions at a rate of $100 per hour. We estimated three data collection events per year and time needed to process and analyze data was assumed to increase over time due to the increased volume of data.

**Table 6:** Partial budget analysis.

Year	Expenditures	# of units	Unit	Price/cost	Total
0	EID tag reader	2	unit	$2,500	$5,000
0	Autodrafter and scale	1	unit	$13,800	$13,800
0	EID tags–ewes	3000	head	$1	$3,000
0	EID tags—replacements	600	head	$1	$600
1	EID tags—replacements, year 1	600	head	$1	$600
2	EID tags—replacements, year 2	600	head	$1	$600
3	EID tags—replacements, year 3	600	head	$1	$600
4	EID tags—replacements, year 4	600	head	$1	$600
0	EID tag applicator	4	unit	$30	$120
0	Sheep flip chute	1	unit	$3,000	$3,000
0	Management software, year 0	3000	head	$0.75	$2,250
1	Management software, year1	3000	head	$0.75	$2,250
2	Management software, year 2	3000	head	$0.75	$2,250
3	Management software, year 3	3000	head	$0.75	$2,250
4	Management software, year 4	3000	head	$0.75	$2,250
0	Incr mgmt time (6 hrs/event)—yr 0	18	hrs	$100.00	$1,800
1	Incr mgmt time (6 hrs/event)—yr 1	18	hrs	$100.00	$1,800
2	Incr mgmt time (7 hrs/event)—yr 2	21	hrs	$100.00	$2,100
3	Incr mgmt time (8 hrs/event)—yr 3	24	hrs	$100.00	$2,400
4	Incr mgmt time (9 hrs/event)—yr 4	27	hrs	$100.00	$2,700
1	Increased labor, year 1	5	days	$172.25	$861
2	Increased labor, year 2	10	days	$186.50	$1,865
3	Increased labor, year 3	15	days	$188.85	$2,833
4	Increased labor, year 4	20	days	$219.05	$4,381
				**Total:**	**$59,910**
	**Increased Income**	**# of units**	**Unit**	**Price/cost**	**Total**
2	Increased lamb crop—year 2 (100%)	15,000	kgs	$3.25	$48,750
3	Increased lamb crop—year 3 (110%)	30,000	kgs	$3.25	$97,500
4	Increased lamb crop—year 4 (120%)	45,000	kgs	$3.25	$146,250
				**Total:**	**$292,500**
	**Cost Savings**	**# of units**	** **	**Price/cost**	**Total**
1	$ on herd health—year 1	600	head	$15	$9,000
2	$ on herd health—year 2	1200	head	$15	$18,000
3	$ on herd health—year 3	1800	head	$15	$27,000
4	$ on herd health—year 4	2300	head	$15	$34,500
1	Worker’s compensation—year 1	1	unit	$15,000	$15,000
2	Worker’s compensation—year 2	1	unit	$30,000	$30,000
3	Worker’s compensation—year 3	1	unit	$45,000	$45,000
4	Worker’s compensation—year 4	1	unit	$60,000	$60,000
				**Total:**	**$238,500**
				**Net Profit:**	**$471,090**

To calculate weight of lamb weaned per ewe exposed we assumed an increase in twins, but this metric does not necessarily indicate twins, heavy singles also result in an increase of weaned weight. Increased twinning rates in ewes increases labor demand. We calculated an increase in labor of five days in year one, 10 days in year two, 15 days in year three, and 20 days in year four. Due to the prevailing labor rates in California, the price/cost of $172.25 was used to represent one day’s pay for one worker. Therefore, an increase in five days of labor could mean one worker for five additional days, five workers for one extra day, or any combination in between. We assumed that income from any selective breeding decisions to increase weight of lamb weaned would not be observed until year two of the budget analysis. In year two, we modeled an increased lambing rate of 10%, making lamb production 100% or a total of 3,000 lambs. This indicates an additional 300 lambs were born in year two. We assumed an average weaning weight of 50 kilograms (110 pounds) per lamb. Lambs are typically marketed at weaning, so in this example the sheep producer would have produced an additional 15,000 kilograms of lamb. We assumed the lambing rate would continue to increase by 10% in years three and four, for an additional 30,000 kilograms of lamb in year 3 and an additional 45,000 kilograms of lamb marketed in year four. Lambs were sold at the United States national 10-year average price of $3.25 per kilogram ($1.48 per pound).

As part of the breeding program, ewes who required treatment for illness, such as footrot or mastitis, received a code in the EID reader to track treatment. Over the course of the four years, culling decisions focused on reducing dollars spent on flock health by removing these individuals from the flock. Using the 20% replacement rate noted above, dollars saved were calculated at $15 per head, with 600 less ewes needing treatment in year 1 and 1,200 less ewes needing treatment in year 2, and so on. The purchase of the sheep flip chute resulted in a substantial increase in worker safety and as a result worker’s compensation insurance dropped by 50% from $120,000 to $60,000. We spread the decrease in worker’s compensation insurance across the four years and gradually increased the dollars saved over the four years of the budget analysis.

The partial budget showed expenditures of $59,910, $292,500 increased income, and cost savings of $238,500 resulting in $471,090 net profit over the period considered. There was no incidence of decreased income to report.

## Discussion

The number of sheep in the United States has steadily declined from 56 million animals in 1942 to approximately 5 million sheep today [15, 16]. In 2021, lamb imports accounted for nearly 70% of lamb consumed in the United States. If sheep numbers continue to decline, there is concern that the American sheep industry may reach a point at which sheep numbers will no longer support key pieces of the industry’s infrastructure. In 2014 the American Lamb Board published a roadmap for the American sheep industry highlighting opportunities, challenges, and a path forward [17]. One response to declining lamb production called for an increase in lamb production with remaining ewes and to promote widespread producer use of quantitative genetic selection. Genetic testing combined with the use of EIDs offers an approach to provide the data required to improve both health management and genetic selection to increase individual animal performance and improve the productivity of flocks.

The use of EIDs in combination with genetic testing, allowed for the collection of individual progeny performance data. This provided insight into sire performance, and identified prolific sires that were also producing lambs with superior carcass merit. The results from the genetic testing in the current study revealed variation in sire prolificacy ranging from 0–135 lambs per sire. Comparable variability was observed in a similar study in the United States called “The Mickel Project”, funded by the American Sheep Industry Association [18]. In that study with 42 Suffolk rams, twelve sired 10 or fewer progeny with two having none, and seven sired more than 55 lambs with two siring over 100 lambs. These authors wrote, “*such variability in ram serving capacity deserves much greater study*.” An important issue to be overcome for genomic technologies to be used in the sheep industry is the time lag between sample collection to usable information being provided to producers. Ideally, parentage information would be returned within a window that facilitates selection prior to ram turnout for the next breeding season.

Table 4 summarizes the average carcass data of ranches A and B, whereas Table 5 shows the carcass performance variability among the progeny of rams with at least 5 offspring. The individual ram data reveals considerable heterogeneity in the sire family deviations as compared to the group average. This variation is hidden in the absence of individual animal performance records. In Fig 3, it can be seen that some sires were producing offspring with significantly more salable meat. This information could be used by producers to keep superior rams and their offspring for future breeding seasons. However, this would only be beneficial to the producer if pricing systems exist that reward selection for these desirable carcass traits. A considerable complication in using progeny carcass data is the unavoidable delay between ram turnout and the collection of necessary data. This limits the practical utility of this strategy and reinforces the value of having EBV/GEBVs available at ram purchase to select genetically superior rams.

Although some US sheep producers have made genetic gains by using EBVs to inform selection decisions, widespread adoption of this technology has been limited in the US sheep industry. The US National Sheep Improvement Program (NSIP) began calculating within‐flock EBVs for ewe reproduction, lamb body weight, gastrointestinal nematode resistance, and wool traits, and since the mid-1990s has been making across-breed EBVs available to participating breeders [19]. As of 2020, there were 294 flocks enrolled in NSIP in 39 US states including the following breeds as a percentage of enrolled flocks: Katahdin (26.9%), Polypay (17.7%), Suffolk (11.2%), Targhee (9.9%) and Rambouillet (6.5%) [1]. Currently, most of the more than 100,000 US sheep producers do not use EBVs to select their rams, and despite the proven benefits of EBVs in genetic improvement in other species, there has been limited uptake of data-driven EBVs to make ram selection decisions on Western range operations.

There are efforts to develop genomic EBVs for American sheep breeds and incorporate genotypic and phenotypic data from medium and high-density SNP arrays into GEBV [1]. One of the key requirements for genomic selection is a large reference population, with dense phenotyping on animals that are genetically related to the wider population to link the genotypic information with the phenotype [7]. To get accurate crossbred GEBVs given the genetic diversity that exists in the commercial sheep population (Fig 1) will require the collection of phenotypes and genotypes from a large number of individuals from the different breeds typically seen in range production systems. GEBV were released for weight and fecal egg count traits for U.S. Katahdin sheep through NSIP starting October 2021. They were based on 5,000 genotypes derived from the Neogen GGP 50k Ovine array [20, 21]. Genomic information improved predictive ability for both traits by as much as 10%, and reduced bias in the evaluation of fecal egg counts. Accuracies of EBV improved by as much as 1.81 times in younger genotyped animals.

Ideally, genotypes and phenotypes collected from commercial sheep such as those collected in this study would be reported into national genetic evaluation schemes to help develop and improve the accuracy of the genomic prediction of EBVs, as is done in several countries. Fig 1 shows the genetic variation that exists between the different flocks enrolled in this study, illustrating the use of different breeds and crossbreeding strategies. Developing accurate GEBVS for crossbred populations with little genetic relationship requires particularly large reference populations. It would be especially helpful to obtain automated carcass data, such as that recorded by the camera in this study, to record objective measurements on valuable commercial traits that are incorporated into ram EBVs. As can be seen in Fig 3 there can be an $80 spread in the sire group average of edible product from different sires. Currently there is no pricing system that rewards carcass quality or yield of salable product, as lambs are predominantly marketed by weight. This market failure means there is no incentive to producers to select for carcass traits despite the considerable value that selection for these traits have to the holistic sheep industry.

The Flock54_SM_ SNP panel reported some markers associated with fertility and disease. The OPP virus causes chronic inflammation of pulmonary and mammary tissues leading to fibrosis of these organs over time causing loss of function (i.e. poor pulmonary gas exchange and reduced milk production). Haplotype variants within the *TMEM154* gene have been reported to impact lifetime susceptibility to OPP virus infection in naturally exposed ewes [22]. Additionally, a small insertion/deletion variant near ZNF389 showed a consistent association with proviral load in three flocks [23]. The OPP resistance scores in Flock 54 uses a susceptibility score on a scale 1–10 based on genotypes at these two loci. There were variability in the scores observed in lambs in this study ranging from 1–8, offering an opportunity to select against animals with a low score indicating increased susceptibility. Electronic ID of individual animals alone could also provide the added benefit of enhanced traceability for OPP prevention and management. Parsons et al. [24] used EIDs to track OPP in two Western range flocks. Ewes that tested positive were kept in a separate band from ewes that tested negative, and replacements were only kept from the OPP negative bands. OPP was eliminated from the two flocks and death loss was reduced by 10% for an estimated cost benefit of $12 per ewe. This impact does not account for the impact of low lamb survival/vigor and increased bottle lambs that often result from OPP.

Susceptibility of sheep to scrapie is influenced by the *PRNP* (prion protein gene) genotype for codons 136, 154, and 171. Three polymorphic codons (136 A (Alanine)/V (Valine), 154 R (Arginine)/H (Histidine), and 171 Q (Glutamine)/R/H) in sheep *PRNP* are related to scrapie resistance/susceptibility status. Alanine, arginine, and arginine at codons 136, 154, and 171 (ARR), respectively, are associated with protection against classical forms of scrapie. In contrast, the PRNP protein variants (VRQ or ARQ) are associated with susceptibility [25]. The genotype reported in the Flock 54 is for the 171 codon. Selection for the R allele and against the Q allele would reduce flock susceptibility to scrapie. Many countries have genotype-based eradication programs that emphasize using rams that express arginine at codon 171 in the prion protein, which is associated with resistance to the classical scrapie agent. The US scrapie eradication program has decreased the number of sheep that are scrapie positive at slaughter by 90% [26]. It should be noted that US Meat Animal Research Center is using knowledge of these *TMEM154* and *PRNP* disease resistance genetic variants to develop a Composite IV line of sheep that includes favorable variants for improved OPP and scrapie disease resistance. This line will additionally combine the coat-shedding, prolificacy, and maternal ability of Katahdin (25%), White Dorper (25%) and Romanov (50%) breeds [1].

Various major genes have been reported to affect prolificacy in sheep, including three related oocyte-derived components, namely, bone morphogenetic protein receptor type 1B (*BMPR1B*), known as *FecB* on chromosome 6 [27], also known as the Booroolla fecundity gene; *GDF9* known as *FecG* on chromosome 5 [28]; and bone morphogenetic protein 15 (*BMP15*), known as *FecX* on chromosome X [28, 29]. There are multiple SNPs in *GDF9* [30], and one of these, known as G8, results in a serine to phenylalanine change at residue 395. This change replaces an uncharged polar group with a nonpolar group at residue 77 of the mature coding region. While the increased ovulation rate seen in heterozygous G8 (*FecGH*) carriers would benefit twinning, sterility has been observed in G8 (FecGH) homozygous animals [28]. For Flock54_SM_, the results are reported on the *FecG*^*V*^ allele of GDF9 [31] which is a missense mutation (Arginine->Cysteine) at amino acid 315. The heterozygote is reported as positive, and homozygote as positive2. It was reported that *FecG*^*V*^ heterozygotes had higher ovulations rates averaging 2.1 +/- 0.1 when compared to 1.2 +/- 0.1 in wild-type ewes (P<0.001) in Ile de France ewes in Brazil [31]. In our study 35 lambs came back with a positive *FecG*^*V*^ result. Six of these lambs came from Ranch A and twenty-eight came from ranch D. One lamb came back with a Positive2 result on Ranch E. However, it should be noted that homozygote ewes for this *FecG*^*V*^ allele are infertile.

The adoption of EIDs by commercial sheep producers has been low, despite this technology being available for over 10 years. Utilizing EIDs alone does not result in production gains. This technology enables the implementation of strategies for achieving desired objectives that may otherwise be prohibitively difficult or time-consuming. Before we began this project, there were varying levels of EID use and IT knowledge among the cooperating producers, as shown in Table 1. Ranch A had been tagging their sheep with EIDs but was not using them to collect data on their flock. Ranch B successfully used EID technology to collect information on foot rot, pounds of lamb per ewe exposed, and wool microns. According to the producer this enabled a more than 75% reduction in the incidence of foot rot, and a 30% increase in lamb crop. Ranch C, a small flock producer, was already tracking individual animal performance data on their flock and had achieved progress towards their identified goals. Switching to EIDs saved them time and labor as compared to the previous system that required 3 types of ear tags and moving the lambs through the chute twice at weaning. Ranch D was interested in the technology but had not yet invested in EIDs. Ranch E used only scrapie tags on a small portion of their flock; all flock production was tracked with paint brands or ear marks. The value of EIDs was seen on both Ranch B and Ranch C when the individual animal performance data was used to make better-informed management decisions towards clear goals.

Producer use of EID technology in our study mimics a peer-reviewed study of commercial sheep farmers from England and Wales, countries where EIDs are mandatory for all sheep. The authors found that farmers who perceived the EID-related technology to be “useful and practical” were more likely to adopt it and the time spent by a farmer in managing the flock in the previous year was positively and significantly associated with adoption of EID technology. Although use of EIDs was mandatory, adoption of EID strategies to improve flock performance remained low as only 53% of farmers owned EID readers to scan the tags, and of these, only 21% were actually using the EID technology for management purposes [3]. There was a sentiment among those farmers not using the EIDs that they were under “external pressure” to adopt the technology and viewed it as an extra bureaucratic burden with no clear benefits. Farmers who had better IT knowledge and understanding of cell phone technology were more likely to use the tags for management purposes. Morgan-Davies [5] found that one farm in Scotland was able to decrease anthelmintic use 46% and labor 36% by utilizing EIDs combined with a digital weighing crate to track individual lamb weights, and treat lambs with anthelmintics only when they did not gain sufficient weight.

Interestingly, and similar to the foot rot example in Ranch B, the use of EID technology for flock management in the UK study was significantly associated with lower lameness levels. England and Wales farmers who typically treated lame sheep with antibiotics were also significantly more likely to adopt EID technology, suggesting they may have positive perceptions of the technology due to its potential to improve health and welfare benefits. Collecting data is only the first part of implementing EID to improve selection decisions. The ability to manage and analyze the data to generate actionable information requires a reasonable working knowledge of computers and data management, especially if there are few commercial service providers to help manage the data collection and analysis process. Another common challenge is the logistics of using EID systems in the field. If a management decision is going to be made chute-side then the information needs to be easily and quickly accessed chute-side.

An increased cost of labor in California means producers are keenly aware of all time spent by herders and herder tasks. A common question raised by producers not using EID asked if the time invested in placing EIDs will pay off in the long run regarding either labor savings, or increased returns to the operation. Producers were hesitant to adopt new practices with a significant up-front cost when they are unsure of returns and how the new practice will fit with their operation and replace multi-generational practices. The partial budget analysis we completed yielded a net return of $417,090 illustrating a greater than 7:1 return on investment within a five-year time frame when investing in an EID system. Although this was a hypothetical study, it aligns with other similar findings. An Australian study modelled the long-term gain, as measured over five years, that can be achieved through changing management decisions based on the data generated using EID and associated technologies (e.g. pregnancy scanning, fleece weighing) on commercial sheep enterprises [32]. The average cost benefit was found to be a $4.12 return for every dollar invested in using EID to improve breeding and selection decisions across Merino and crossbred/composite type enterprises. In that study the greatest benefit was achieved through culling dry animals via pregnancy scanning as this effectively assisted to increase the overall reproductive rates of the flock.

The Cooperative Research Centre for Sheep Industry Innovation (Sheep CRC) in Australia has done a number of case studies looking at the value returned from EID systems [33]. In their studies, the first step is identifying breeding objectives and applicable traits to measure. Describing the breeding objective helped identify the traits that would drive profit in the business and hence what should be measured in the flock. One farm in Australia increased lambing percentage 20% by tracking and culling twice-dry ewes. Another analysis calculated a 10% increase in weaning rate could raise profit per hectare by 14–24%. In another study [34], net reproductive rate increased 6% on average in flocks where ewes were tracked individually and ewes that failed to produce a lamb two years in a row were culled. Dickson [35] used EIDs to track kilograms of lamb produced per ewe and highlighted average size adult ewes by weight that produced above average kilograms of lamb, effectively identifying the high-performing ewes in the flock. By identifying and culling the lowest performing ewes they found that net profitability of each ewe could be increased $25.47 over her four-year lifetime. At the conclusion of that study the author recommended,

*“Significant opportunities exist to increase profitability through identifying the performance of individual animals within a flock*. *However*, *before embarking on this process it is critical to have a clear plan and objective for conducting this data collection*. *Sheep producers should have a breeding objective that details their flock production targets which will assist in determining the required information to be collected*. *Once the data that is necessary to be collected is identified*, *then appropriate equipment and processes can be investigated*. *It is critical that all data collected is utilized in a decision making process as there is a cost involved in any form of data collection*.*”* [35].

There are also potential downstream applications of EID to support tracing food from farm to fork [36]. Block chain management offers further opportunities for sheep producers by providing consumers with information on where and how their food was produced while they are at the meat counter. Consumers are increasingly interested in knowing where their food comes from, and with less than 1.5% of the population directly involved with or exposed to food production, supporting consumer access to information about food production practices provides an important outreach opportunity for sheep producers. For example, a consumer could scan a QR code on a package and be directed to a website where they can learn about the sheep producer who raised the lamb in their hand, creating a connection between producer and consumer. In this case, EID could be seen to inform the customer base about attributes of the product such as well-being of animals or product quality or origin, rather than a tool to increase production [37].

It is really through the integration of individual performance data, DNA testing and genetic evaluations that the synergistic value of these emerging technologies becomes realized. At the time of writing, the Flock54_SM_ test is listed at a price of $20/head. As a stand-alone test this is difficult to recoup through paternity information and ad hoc within‐flock commercial ranch genetic evaluations alone. However, when this information is combined with individual performance record collection enabled by EID and these data are then fed into a national database, the value of this information is increased as it provides data to increase the accuracy of the EBVs that can be developed by NSIP. Several countries have set up reference populations to facilitate the accuracy of GEBVs and GS in their sheep populations [38]. For many sheep breeds, the main obstacle is to build a suitable reference population [39]. For example, in US Suffolk it was estimated that for weaning weight in Suffolk, using a heritability of 0.15 as determined by NSIP, reference population sizes of 250, 3000, and 10,000 would result in a baseline GEBV accuracy values of 0.116, 0.371, and 0.578, respectively, for animals with no direct relatives in the reference population or phenotype for the trait of interest [40]. A substantial increase in the accuracy of GEBV in small sheep breeding programs may be achieved through the use of very low-density genotyping panels, such as Flock54_SM_, combined with an imputation processes that employs medium density (e.g. 50K SNP) arrays from related individuals [39]. As observed with genomic prediction accuracy, imputation accuracy increases as close relatives and pedigree information are included in the reference population. With strategic medium density genotyping of key influential individuals in the pedigrees, the accumulation of phenotypic and genotypic data such as those collected in this study could therefore provide benefits beyond those derived by the individual producer alone, as it could aid efforts to develop GEBV and data-driven selection programs to allow the US sheep industry to accelerate the rate of genetic improvement.

## Conclusions

The uptake of EIDs and DNA testing by commercial Western range sheep producers has been low, despite the availability of these technologies for over a decade. Jointly, genetic testing combined with the use of EID offer an approach to provide the individual animal performance and parentage data required to improve flock health and reproductive management and enable genetic selection for more productive and profitable individual animals. In this demonstration project producers obtained varying levels of useful information from the implementation of EID. Producers who tracked individual animal performance observed positive changes in the overall health and productivity of their flock which led to marked financial benefits. Those that tracked animals through slaughter and received carcass data have a greater opportunity to derive value from EID. The collection of individual progeny carcass data provided insight into sire performance, providing for the within-flock identification of prolific sires that were producing lambs with significantly more saleable meat as compared to their flock mates. There was as much as an $80 difference in the average edible product from camera-graded carcasses derived from lamb groups sired by different rams. Ideally, all this data from commercial animals would flow into national genetic evaluations, such as the National Sheep Improvement Program, to help develop and improve the accuracy of the GEBVs. This would help facilitate widespread commercial sheep producer use of data-driven quantitative genetic selection as recommended in the roadmap for the American sheep industry based on GEBVs, as has been adopted in several major sheep producing countries.
